# Water mites (Acari, Hydrachnidia) of riparian springs in a small lowland river valley: what are the key factors for species distribution?

**DOI:** 10.7717/peerj.4797

**Published:** 2018-05-24

**Authors:** Andrzej Zawal, Robert Stryjecki, Edyta Buczyńska, Paweł Buczyński, Joanna Pakulnicka, Aleksandra Bańkowska, Tomasz Czernicki, Katarzyna Janusz, Agnieszka Szlauer-Łukaszewska, Vladimir Pešić

**Affiliations:** 1 Department of Invertebrate Zoology and Limnology, Institute for Research for Biodiversity, Centre of Molecular Biology and Biotechnology, Faculty of Biology, University of Szczecin, Szczecin, Poland; 2 Department of Zoology, Animal Ecology and Wildlife Management, University of Life Sciences in Lublin, Lublin, Poland; 3 Department of Zoology, Maria Curie-Sklodowska University in Lublin, Lublin, Poland; 4 Department of Ecology and Environmental Protection, University of Warmia and Mazury in Olsztyn, Olsztyn, Poland; 5 Department of Biology, University of Montenegro, Podgorica, Montenegro

**Keywords:** Inundation, Permanence, Crenotypic species, Landscape factors, Synecological groups

## Abstract

This paper examines the impact of disturbance factors—flooding and intermittency—on the distribution of water mites in the riparian springs situated in the valley of a small lowland river, the Krąpiel. The landscape factors and physicochemical parameters of the water were analysed in order to gain an understanding of the pattern of water mite assemblages in the riparian springs. Three limnological types of springs were examined (helocrenes, limnocrenes and rheocrenes) along the whole course of the river and a total of 35 water mite species were found. Our study shows that flooding influences spring assemblages, causing a decrease in crenobiontic water mites in flooded springs. The impact of intermittency resulted in a high percentage of species typical of temporary water bodies. Surprisingly, the study revealed the positive impact of the anthropogenic transformation of the river valley: preventing the riparian springs from flooding enhances the diversity of crenobiontic species in non-flooded springs. In the conclusion, our study revealed that further conservation strategies for the protection of the riparian springs along large rivers would take into account ongoing climatic changes and possible the positive impact of the anthropogenic transformation of river valleys.

## Introduction

Hydrachnidia (water mites) are the most important group of freshwater arachnids and a robust component of macroinvertebrate assemblages in spring habitats, both in terms of abundance and species richness (Di Sabatino et al., 2008). Compared with other biotic groups that inhabit springs, Hydrachnidia include the highest percentage of true crenobiontic species (Smith, 1991; Gerecke et al., 1998; Buczyński et al., 2003; Gerecke & Di Sabatino, 2007). A review of the literature from the last six years (Web of Science Database, 2011–2016 topic ‘Hydrachnidia’) shows that just only six of the 132 published papers were devoted to research on water mites in spring ecosystems (Bottazzi et al., 2011; Goldschmidt & Melzer, 2011; Stoch et al., 2011; Cantonati et al., 2012; Martin & Brunke, 2012; Pešić et al., 2016)

Little research has been done on the impact of disturbance factors such as flooding and intermittence on spring assemblages. Von Fumetti & Nagel (2012) stated that the impact of disturbance events such as floods is underestimated. Recently, Von Fumetti, Dmitrović & Pešić (2017) showed that flooding significantly influences the composition of the species assemblages of the riparian springs in the valley of the River Cvrcka in Dinaric karst, leading to a higher proportion of rhitrobiontic (and a smaller percentage of crenobiontic) taxa in the flooded springs. On the other hand, some studies have shown that the water mite composition inhabiting intermittent springs differs from those assemblages that inhabit perennial springs (Smith, Wood & Gunn, 2003; Wood et al., 2005). To date, the influence of neither of these disturbance factors on water mite assemblages in riparian springs along a lowland river has been studied. Those water mite species typical of springs have a susceptible dispersal ability, and therefore the damaged fauna recovers very slowly (Gerecke, Martin & Gledhill, 2017). The presence in the flooded spring of this type of fauna indicates one benefit of the impact of spring waters over flood waters.

Water mites are also a very useful, though neglected, group of animals with bioindication properties (Więcek, Martin & Lipiński, 2013; Goldschmidt, 2016). They can be used both as bioindicators of the habitat structure of reservoirs and their productivity and pollution (Kowalik & Biesiadka, 1981; Biesiadka & Kowalik, 1991; Cicolani & Di Sabatino, 1991; Zawal, 1992; Zawal, 1996; Martin & Brinkmann, 2003; Martin & Brunke, 2012; Zawal et al., 2013). The species of water mites associated with springs, due to their particularly high stenotypism, are extremely sensitive to environmental changes; thanks to this, their bioindication value is very high (Biesiadka & Kowalik, 1999; Di Sabatino et al., 2008; Goldschmidt & Melzer, 2011; Martin & Brunke, 2012).

Previous studies of the Krąpiel valley showed environmental factors to be acting at different levels of organization in the environment, i.e., (1) the landscape level, (2) the macrohabitat level and (3) the mesohabitat level; they all affect the species composition and the abundance of water mite assemblages (Stryjecki et al., 2016; Buczyńska et al., 2017; Zawal et al., 2016b; Zawal et al., 2017).

The present study examines the influence of disturbing factors and environmental parameters on the fauna of riparian springs. The following questions were addressed in the study:

1. How do disturbance factors such as flooding and intermittency affect the water mite assemblage in riparian springs?
2. How do physicochemical and the landscape parameters involved influence water mite assemblages of riparian springs?

## Materials & Methods

### Study sites

Descriptions of the study area in general and the River Krąpiel in particular as well as the water bodies in the valley, are given in Stryjecki et al. (2016) and Zawal et al. (2017).

**Figure 1:**
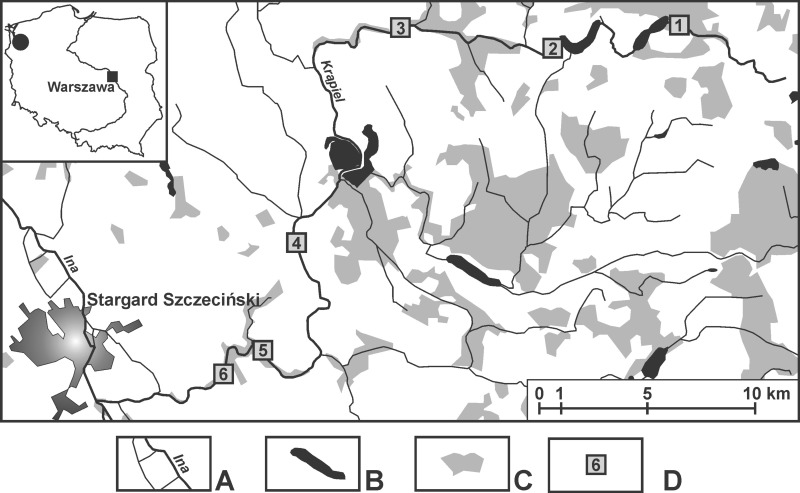
Location of the sampling sites. (A) Rivers. (B) Lakes and fish ponds. (C) Forests. (D) Localities (Z1–Z6).

The samples were taken from springs in the valley of the small (about 60 km long) lowland River Krąpiel (in north-western Poland) (Appendix S1). The research covered the entire length of the river, and the distance between localities was in the range of 4.5–15 km. Six localities were chosen: Z1 (53°28′10.63″N 15°21′41.79″E), Z2 (53°27′36.97″N 15°16′33.2″E), Z3 (53°27′41.47″N 15°12′22.94″E), Z4 (53°21′6.4″N 15°11′5.23″E), Z5 (53° 20′29.56″N 15°9′15.04″E), Z6 (53°19′58.14″N 15°7′57.54″E); along the valley where springs occurred in the greatest numbers (Fig. 1). The springs at one locality were in close proximity, less than 50 m apart, and shared the same springbrook (Table 1). The number of springs examined was based on the spatial differentiation of each particular locality and was as follows: two springs at locality Z4, three at locality Z1 and four each at localities Z2, Z3, Z5 and Z6. For each spring, the dominant sediment types, the surrounding and submerged vegetation, its permanent or temporary flow, the depth and distance from the river, as well as the inundation/non-inundation status were all documented (Table 1).

**Table 1:** List of localities and characteristics of the springs. (Z/s) Locality no./spring no. (Dist) Distance from the river [m]. (Type) Spring type: H, helocrene; L, limnocrene; R, rheocrene. (Inun) Inundation: C-I, catchment inundation; R-I, river inundation; No I, no inundation. (Perm) Permanence: P, permanent spring; Non-P, non-permanent spring.

Z/s	Type	Depth [m]	Area [m]^2^	Dist [m]	Inun	Perm	Surroundings	Bottom	Vegetation/ remarks
Z1/1 *N* = 3	L	0.5	5	10	C-I	Non-P	alder carr	silt, leaves	
Z1/2 *N* = 3	L	0.4	5		C-I	Non-P		silt	*Carex acutiformis*
Z1/3 *N* = 1	H	0.01	2		C-I	Non-P		silt, leaves	*Cardamine amara*
Z1/4 *N* = 4	H	0.1	1	1	R I	P		silt, leaves	no water mites
Z2/1 *N* = 4	R	0.1	1	20	No I	P	alder carr, willow thickets	sand, silt, leaves	
Z2/2 *N* = 4	H	0.01–0.02	1		No I	P		silt, leaves	sedges, mosses
Z2/3 *N* = 3	H	0.01–0.02	2		No I	Non-P		silt, leaves	sedges, *Cardamine amara*
Z2/4 *N* = 4	H	0.01–0.02	3		No I	P		leaves	sedges, *Cardamine amara*
Z3/1 *N* = 4	R	0.01	1	50	No I	P		gravelly-silty	no water mites
Z3/2 *N* = 4	H	0.02	3	50	No I	P		silt, leaves	
Z3/3 *N* = 4	R	0.02	1	50	No I	P		sandy-silty	
Z3/4 *N* = 1	H	0.02	4	3	R-I	Non-P		silt, leaves	*Cardamine amara*
Z3/5 *N* = 1	H	0.01	4	50	No I	Non-P		silt, leaves	
Z4/1 *N* = 3	H	0.01–0.02	2	50	No I	Non-P	alluvial forests with *Alnus glutinosa* and *Fraxinus excelsior*	silt, leaves	sedges
Z4/2 *N* = 4	H	0.02	3	3	R-I	P		mud	*Typha latifolia, Carex acutiformis*
Z4/3 *N* = 2	H	0.1	2	50	No I	Non-P		silt, leaves	*Cardamine amara,* no water mites
Z5/1 *N* = 4	R	0.05	1	15	No I	P	oak-hornbeam stands	gravelly-silty	
Z5/2 *N* = 4	H	0.01	1	10	No I	P		stones	
Z5/4 *N* = 4	H	0.02	2	10	No I	P		sandy-silty, leaves	
Z5/5 *N* = 1	R	0.05	1	3	No I	Non-P		silt, leaves	grasses
Z6/1 *N* = 2	H	0.01	5	3	R-I	Non-P	alder carr	silt, leaves	sedges, *Cardamine amara*
Z6/2 *N* = 4	H	0.05	5	10	R-I	P		silt, leaves	sedges
Z6/3 *N* = 4	L	0.1	2	2	R-I	P		silt, leaves	sedges
Z6/4 *N* = 4	L	0.2	4	3	R-I	P		leaves	*Carex acutiformis, Petasites* sp.

### Faunistic sampling

In the year of research, the River Krąpiel was characterized by an average water level, which means that the degree of flooding or drying of the valley across particular seasons was one of the most frequent found in long-term observations (A Zawal & A Szlauer-Łukaszewska, pers. obs., 2008–2012). The samples were taken during the floods and after they dissipated, with the exception that, in the case of complete drying out, the spring of the samples were not collected.

The samples were taken in May, July, September and November 2011, but the springs were monitored continuously over the seven-month period (May–November) in order to assess whether they were not flooded and were not dried up. Owing to the small size of these springs and the risk of destroying them, only one sample was taken from an area of about 0.25 m^2^ with a hand net at each spring. A total of 76 samples were collected (one sample from each spring, 4 times a year) (Table 1). An inherent feature of the springs was their very small surface area, which resulted in unusually low numbers of water mites there. For this reason, the material collected should be treated as a ‘general population’ rather than as a statistical sample from this population. Therefore, despite the unusually low number of individual samples, further statistical analysis is justified. On the other hand, we should be very cautious in extrapolating the conclusions drawn from this analysis to other research areas.

### Environmental parameters

A hydrological assessment of the river valley was done for each of the localities (Z1–Z6) using the standard River Habitat Survey (RHS) method, a technique ensuring that the results are comparable with those of other studies (Szoszkiewicz & Gebler, 2012). The RHS methodology was modified somewhat for the purposes of this study: assessments were made for stretches of 100 m rather than the standard 500 m length of river channel. The fieldwork enabled the following indices to be calculated (Szoszkiewicz & Gebler, 2012): the habitat modification score (HMS), the habitat quality assessment (HQA), the river habitat quality (RHQ) and the river habitat modification (RHM) indices.

The landscape structure analysis was based on buffer zones and catchment areas delineated for each locality (Z1–Z6). Each buffer zone was taken to be a circle of a radius of 500 m around the point on the river defining the locality. Analysis of the spatial structure of the buffer zones and catchment areas was based on a set of landscape metrics calculated using TNTmips software by MicroImages. The classification was based on data from Landsat TM7 28-05-2003. Land cover classes were determined according to the Corine classification (European Environment Agency, 2007). Buffer zones with a radius of 500 m from the sampling point were marked out using GPS. The following measures and indices were used to analyse the landscape structure (abbreviations in brackets): 1. measurements of patch area—area (AREA); 2. measurements of patch density and size: the number of patches (NUMP), the mean patch size (MPS), the median patch size (MEDPS), the patch size standard deviation (PSSD) and the patch density (PD); 3. boundary measurements: the total edge length (TE), the edge density (ED) and the mean edge length (MTE); 4. shape measurements: the mean shape index (MSI), the mean patch fractal dimension (MPFD) and the sum of the patch shape indices (SUM); 5. diversity and distribution indices: the mean distance to the nearest neighbour (MNN), the Interspersion and Juxtaposition Index (IJI), Shannon’s patch diversity index (SDI), the Shannon evenness index (SEI), the size of the catchment area from the sources (a cat cu), the size of the catchment area (a cat), the length of catchment area boundaries, roughness (Ra), contagion (Cr), the river gradient (river fa), distance from source (d source), the area (a) of each patch (forests, fields, swamps, built-up areas, meadows, shrubs, wasteland and water bodies) and the distance from the river (d) of each patch (forests, agricultural areas, swamps, built-up areas, meadows, shrubs, wasteland and water bodies); and 6. the characteristics of particular patches (forests, fields, swamps, built-up areas, meadows, shrubs, wasteland and water bodies) in the buffer zones: the area (CA), the mean patch size: (MCA), the mean shape index (MSI), the patch density (PD) and the ratio of area to boundary length (L/D).

The following environmental parameters were measured at the springs: insolation (insolati %), density of aquatic vegetation (plants, on a scale from 0 to 5, where 0 stands for no plants and 5 indicates total overgrowth by plants), water temperature (temp.° C), water pH (pH), total hardness (hardness mg CaCO_3_/dm^3^), conductivity (cond. µS/cm), solid concentration (mg/dm^3^), oxygen content (O_2_mg/dm^3^), ammonia nitrogen (NH_4_mg/dm^3^), nitrate nitrogen (NO_3_mg/dm^3^), phosphates (PO_3_mg/dm^3^), ferric ions (Fe^3+^ mg/dm^3^), BOD5, proportion of mineral sediment (mineral %), proportion of organic sediment (organic %), mean sediment grain size (M mm) and sediment sorting (W mm). The water parameters, i.e., temperature, pH, electrolytic conductivity and the dissolved oxygen content, were measured using an Elmetron CX-401 multiparametric sampling probe, water flow by using a SonTek acoustic FlowTracker flowmeter, BOD_5_ by Winkler’s method, the other parameters with a Slandi LF205 photometer, and insolation by using a CEM DT-1309 light meter. Three measurements were performed on each sampling occasion and the median was used for further analysis.

### Statistical analysis

On the basis of the literature (Smit & Van der Hammen, 2000; Davids et al., 2006; Biesiadka, 2008; Di Sabatino et al., 2010; Gerecke et al., 2016; Gerecke, Martin & Gledhill, 2017), the water mite fauna was divided into four synecological groups: crenobionts, crenophiles, rheobionts, rheophiles and lenitobionts. The species nomenclature and the systematic layout follow Davids et al. (2006), Di Sabatino et al. (2010) and Gerecke et al. (2016).

The ordering of the springs based on faunistic data was conducted using nonmetric multidimensional scaling (NMDS). PAST version 3.16 software (Hammer, Harper & Ryan, 2001) was used to perform the NMDS analysis: these were done using both Jaccard and Bray–Curtis formulas. In the Bray–Curtis analysis, the image is strongly dominated by sites with very few water mite specimens, so that the other sites are clustered in a small space. These sites are ordered in nearly the same way as in the image produced by the Jaccard analysis, which creates a clearer image. The Jaccard image was therefore used in the subsequent analysis.

Due to the large number of analyzed variables which had a potential impact on the water mite assemblages, Principal Components Analysis and classification (PCA) based on a correlation matrix was applied. The analysis was based on the occurrence of Hydrachnidia in the collected samples.

PCA analysis enabled us to reduce the number of variables to four main components (factors), of which we selected two, corresponding to the greatest eigenvalues (>1.0) and which explained over 80% of the total variance. Variables expressing the characteristics of particular habitat parameters were the active variables, while the analyzed species were the additional ones. The data used for the analysis had previously been log transformed to obtain a normal distribution. The normality of the distribution was tested using the Shapiro–Wilk test.

The significance of the differences in the abundance of particular species of water mites in springs was tested by non-parametric analysis of variance (Kruskal–Wallis) and Spearman’s correlation coefficient. All the calculations were performed using the Statistica 12 programme.

## Results

### General characteristics of Hydrachnidia fauna and synecological groups. Spring inundation and permanence

Altogether 219 water mites belonging to 35 species were identified (Table 2): 45% were collected from four limnocrenes (99 ind.), 35% from 15 helocrenes (77 ind.) and 20% from five rheocrenes (43 ind.).

**Table 2:** Species composition and numbers of water mites collected in springs situated in the Krąpiel valley.

No.	Species	Abr.	SG	Helocrenes (1/3-6/2)													Limnocrenes (1/1-6/4)					Rheocrenes (2/1-5/1)			Total
				1/3	2/2	2/3	2/4	3/2	3/4	3/5	4/1	4/2	5/2	5/4	6/1	6/2	1/1	1/2	5/5	6/3	6/4	2/1	3/3	5/1	
1.	*Eylais hamata* Koenike, 1897	Eyl ham	sb						1																1
2.	*Hydrachna crassipalpis* Piersig, 1897	Hyd cra	sb															3							3
3.	*Hydrachna leegei* Koenike, 1895	Hyd lee	sb															2							2
4.	*Euthyas truncata* (Neuman, 1874)	Eut tru	sb									2													2
5.	*Parathyas barbigera* (K. Viets, 1908)	Par bar	sb															1							1
6.	*Parathyas bruzelii* (Lundblad, 1926)	Par bru	sb															2							2
7.	*Parathyas dirempta* (Koenike, 1912)	Par dir	sb															3							3
8.	*Parathyas pachystoma* (Koenike, 1914)	Par pac	sb	1	9	4		1			4						1	38			8				66
9.	*Parathyas palustris* (Koenike, 1912)	Par pal	kb		4	1		1		1	2					1		6							16
–	*Parathyas* sp. Lundblad, 1926	–	–									4				2		9	1	1					17
10.	*Thyopsis cancellata* (Protz, 1896)	Ty can	kf								1														1
11.	*Hydryphantes ruber* (Geer, 1778)	Hyd rub	sb															7							7
12.	*Hydryphantes tenuipalpis* Thon, 1899	Hyd ten	sb			1												1							2
13.	*Bandakia concreta* Thor, 1913	Ban con	kb				1																		1
14.	*Lebertia glabra* Thor, 1897	Leb gla	rb								1		1											1	3
15.	*Lebertia maglioi* Thor, 1907	Leb mag	rb					1																	1
16.	*Lebertia oblonga* Koenike, 1911	Leb obl	rb																	1					1
17.	*Lebertia salebrosa* Koenike, 1908	Leb sal	rb											1								1	1	5	8
18.	*Lebertia sparsicapillata* Thor, 1905	Leb spa	rb																			1			1
19.	*Lebertia separata* Lundblad, 1930	Leb sep	kb					1																	1
20.	*Lebertia sinuata* K. Viets, 1930	Leb sin	rb																			4			4
–	*Lebertia* sp. Neuman, 1880	–	–								1														1
21.	*Rutripalpus limicola* Sokolow, 1934	Rut lim	kb				1																		1
22.	*Sperchon squamosus* Kramer, 1879	Spe squ	kf		1																	23			24
23.	*Sperchon thienemanni* Koenike, 1907	Spe thi	kf										3									1	2		6
–	*Sperchon* sp. Kramer, 1877	–	–																			2			2
24.	*Hygrobates norvegicus* (Thor, 1897)	Hyg nor	kb																			1			1
25.	*Atractides nodipalpis* Thor, 1899	Atr nod	rb													1									1
26.	*Piona nodata* (Müller, 1776)	Pio nod	sb														1								1
27.	*Piona laminata* (Thor, 1901)	Pio nla	sb															3							3
28.	*Tiphys latipes latipes* (Müller, 1776)	Tip lat	sb														1	2		1					4
29.	*Tiphys scaurus* (Koenike, 1892)	Tip sca	sb		1																				1
–	*Tiphys* sp. Koch, 1836	–	–								1					1					1				3
30.	*Arrenurus fimbriatus* Koenike, 1885	Arr fim	sb		1																		1		2
31.	*Arrenurus conicus* Piersig, 1894	Arr con	kf			1					5									1	1				8
32.	*Arrenurus cylindratus* Piersig, 1896	Arr cyl	kf									1													1
33.	*Arrenurus mediorotundatus* Thor, 1898	Arr med	sb		1		5				2	2			1	1									12
34.	*Arrenurus integrator* (Müller, 1776)	Arr int	sb																		1				1
35.	*Arrenurus pugionifer* Koenike, 1908	Arr pug	sb															1			2				3
–	*Arrenurus* sp. Dugès, 1834	–	–								1														1
	**TOTAL Specimens**			**1**	**17**	**7**	**7**	**4**	**1**	**1**	**18**	**9**	**4**	**1**	**1**	**6**	**3**	**78**	**1**	**4**	**13**	**33**	**4**	**6**	**219**
	**Species**			**1**	**6**	**4**	**3**	**4**	**1**	**1**	**6**	**3**	**2**	**1**	**1**	**3**	**3**	**12**	**1**	**3**	**4**	**6**	**3**	**2**	**35**

**Notes.**

Abbreviation SG Synecological groups kb crenobionts kf crenophiles rb rheobionts sb stagnobionts

(Σ) Sum. (1/1-6/4) Sites within particular localities (see Table 1).

**Figure 2:**
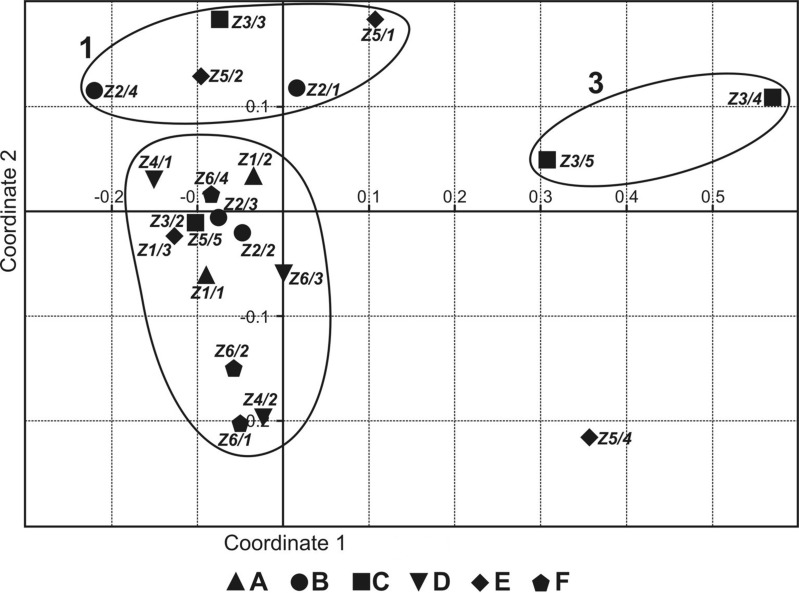
Non-metric multidimensional scaling of faunistic assemblages of the springs surveyed. Localities: (A) Z1; (B) Z2; (C) Z3; (D) Z4; (E) Z5; (F) Z6.

The NMDS ordering (Fig. 2) shows three groups of sites: 1—permanent, non-flooded sites, with crenophilic and crenobiontic species; 2—sites flooded by the river or by water flowing in from the catchment area, partially drying out, with a mixed fauna consisting of species characteristic of standing and astatic water bodies plus a small share of rheophilic species; and 3—highly astatic sites, in which only a few water mite specimens were recorded (Table 2). Site Z5/4 was a specific case, because there was one individual of *Lebertia salebrosa*, species not found anywhere else (Table 2). Coordinate 1 illustrates the gradient of increasingly astatic conditions, from permanent, typical spring sites to sites that are in place for only 2–3 months. Coordinate 2 illustrates the gradient of the increasing impact of floodwaters, from non-flooded, typical spring sites to sites that were flooded for 2–3 months.

The number of species was greatest in the helocrenes (20) followed by helocrenes (16) and the lowest number in rheocrenes (8). The differences in the numbers of individual mites caught in the different types of spring were statistically insignificant (Kruskal–Wallis test: H (2, *N* = 96) = 3,200,929 *p* = 0.2018).

Differences between the abundance of synecological groups were statistically significant (Kruskal–Wallis test: H (2, *N* = 63) = 11,8757 *p* = 0.0026). Lenitobionts (59.6%, 18 spp.) were predominant in all the springs taken together; there were far smaller proportions of crenophiles (20.5%, 5 spp.), crenobionts (10.2%, 5 spp.) and rheobionts (9.7%, 7 spp.) (Table 1). The percentage of lenitobionts was greatest in the limnocrenes (89.7%). The proportion of lenitobionts was also high (55.3%) in the helocrenes, but this type of spring also supported the greatest proportion of crenobionts (19.4%, 4 spp.). The most numerous synecological group in the rheocrenes consisted of crenophiles (63.5%, 2 spp.).

Correlations between the synecological groups and the location of the spring relative to the river (in terms of latitude and height) were not statistically significant. Having said that, positive correlations for crenophiles and crenobionts were obtained in relation to distance (0.427) and height (0.101), negative correlation for rheophilous and rheobionts in relation to distance (−0.095) and positive in relation to height (0.0004). In addition, a positive correlation for stagnophilous species was obtain in relation to distance (0.066) and a negative one in relation to height (−0.253).

The percentage of crenobionts and crenophiles was greatest in non-flooded springs (51.5% vs. 10.2% in flooded springs) (Appendix S2). Lenitobionts were dominant in flooded springs (88.1%) with a small proportion of crenobionts (10.2%) and rheobionts (1.7%) (Appendix S2). Catchment inundation had a much more significant impact on the character of the water mite fauna than river inundation (Appendix S2). In permanent springs, crenobionts and crenophiles together accounted for 44.3% of the water mites (Appendix S3). In springs that periodically dried out, crenobionts and crenophiles together accounted for only 17.3% of the total. More species (24) were recorded in permanent springs than in temporary ones (18 species). A characteristic feature of the temporary springs, besides the large percentage of species typical of temporary water bodies, was the absence of rheobionts.

### The relationship between species and environmental variables

The analysis of the factor coefficients of variables determining the relationships between habitat parameters (active variables) and species (additional variables), for the first two components (factors) shows that both axes corresponding with the greatest own values (axis 1–8.152, axis 2–4.821) explain a total of 81.1% of the total variance. The first axis is strongly correlated with the variables: ‘a forest’ (*r* = 0.9516), ‘a mead’ (*r* = 0.9076), ‘a st water’ (*r* = 0.8610), ‘a shrub’ (*r* = 0.8457), ‘a marsh’ (*r* = 0.9542), ‘a wast’ (*r* = 0.5993), ‘d marsh’ (*r* = 0.9542), ‘d wast’ (*r* = 0.9532) (positive correlations) and ‘L (1)’ (*r* =  − 0.85222), and ‘L (2)’ (*r* =  − 0.9515) (negative correlations). The second axis is strongly correlated with ‘PD (13)’ (*r* = 0.9565) and ‘MSI (13)’ (*r* = 0.9565) (positive correlations) and ‘HMS’ (*r* =  − 0.992), ‘RHM’ (*r* =  − 0.737000) and ‘L (15)’ (*r* =  − 0.917681) (negative correlations).

**Figure 3:**
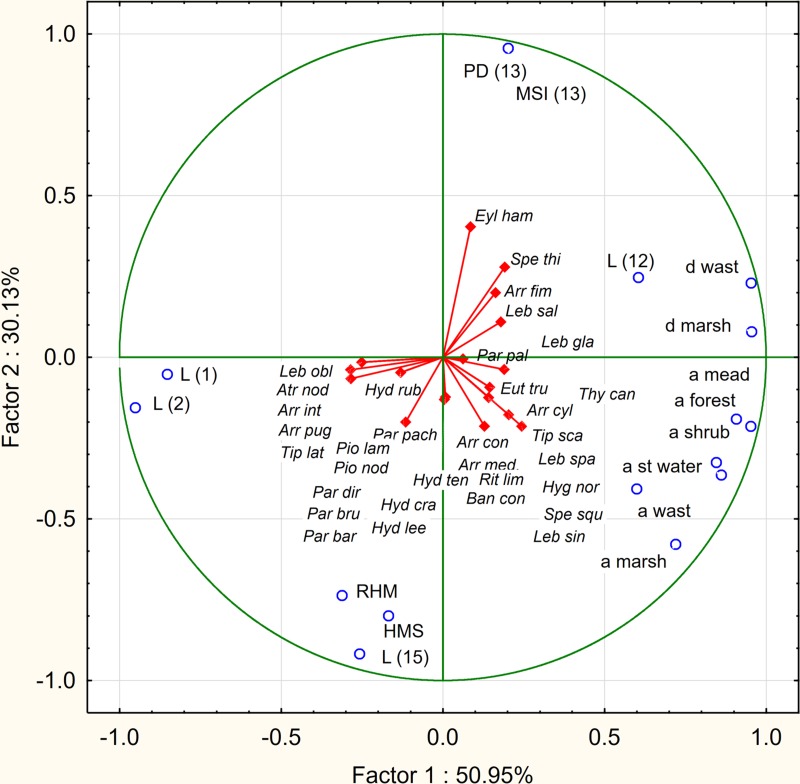
PCA diagram displaying the dependence of water mites on environmental parameters.

The analyzed species form three distinct assemblage in accordance with their ecological preferences. In the second quarter there are hemistenothermic species: the crenobiont (*Sperchon thienemanni*) and the rheobiont (*Lebertia salebrosa*). In the third quarter of the graph there are crenophilous and crenobiontic species (*Arrenurus cylindratus, A. conicus, A. mediorotundatus, Bandakia concreta, Hygrobates norvegicus, Lebertia separata, Parathyas palustris, Rutripalpus limicola, Sperchon squamosus, Thyopsis cancellata*) which create clusters showing positive correlations with: ‘a mead’, ‘a forest’, ‘a shrub’, ‘a wast’, ‘a marsh’, ‘a st wat’, ‘d wast’, ‘d marsh’, ‘PD (13)’, ‘MSI (13)’, ‘L (12)’, ‘L (15)’, ‘RHM’, ‘HMS’; and negative with: ‘L (1)’ and ‘L (2)’. The most eurytopic ecological component creates a cluster in the fourth quarter. These are lenitobiont species (*Arrenurus integrator, A. pugionifer, Hydrasna crassipalpis, H. leegei, Hydryphantes ruber, H. tenuipalpis, Parathyas barbiger, P. bruzelii, P. dirempta, P. pachystoma, Piona nodata, P. laminata, Tiphys latipes*). They are negatively correlated with ‘a mead’, ‘a forest’, ‘a shrub’, ‘a wast’, ‘a marsh’, and ‘st wat’, and positively correlated with ‘RHM’, ‘HMS’, ‘L (1)’and ‘L (2)’. Single eurytopic (lenitobiont) species (*Eylais hamata, Euthyas truncata, Tiphys scaurus, Arrenurus fimbriatus*) are also associated with stenotopic species in the second and third quarters of the graph (Fig. 3).

## Discussion

To date, only a few studies have focused on the macroinvertebrate assemblages of riparian springs (Pešić et al., 2016; Savić, Dmitrović & Pešić, 2017; Von Fumetti, Dmitrović & Pešić, 2017)—they have stressed the absence of eucrenal-hypocrenal zonation (Pešić et al., 2016) and the importance of the main river in the formation of spring species assemblages (Von Fumetti, Dmitrović & Pešić, 2017). All these studies have yielded a relatively small number of water mites in riparian springs, in terms of both taxon diversity and abundance. The riparian springs in the Krąpiel valley are similarly characterized by a small abundance of water mite species: most springs were small with a minimal outflow, adjacent to the river, and so small numbers of water mites in the springs described here were anticipated.

Our study showed that the water mite assemblages of the riparian springs in the Krąpiel valley were dominated by crenoxenes. Most lenitobiontic species were found in limnocrenes, a quite high percentage (>50%) was found in helocrenes, but only a low proportion of crenoxenes (10%) in rheocrenes. Some other studies have shown that different spring habitats differ in their proportions of crenobiontic species: crenobionts are generally dominant in helocrenes and crenophiles in rheocrenes, while a high percentage of crenoxenes is characteristic of limnocrene springs (Biesiadka & Kowalik, 1978; Biesiadka, Cichocka & Warzecha, 1990; Martin & Brunke, 2012).

In the riparian springs along the River Krąpiel, the percentages of crenobiontic and crenophilous species were greatest in helocrenes (32%) and rheocrenes (60%). Three crenobiontic species—*Bandakia concreta*, *Rutripalpus limicola* and *Lebertia salebrosa*—were found in the helocrenes, and six crenophiles and crenobionts—*Sperchon squamosus*, *S. thienemanni*, *Hygrobates norvegicus*, *Arrenurus conicus* and *Thyopsis cancellatus*—were found in the rheocrenes. All these species are well-known species characteristic of helocrenic and rheocrenic springs, respectively, in central Europe (Biesiadka, 1979; Böttger & Martin, 1995; Martin, 1996; Van der Hammen & Smit, 1996; Biesiadka, 2008; Di Sabatino et al., 2010).

In previous research (Von Fumetti, Dmitrović & Pešić, 2017), the relationship between the type of fauna inhabiting riparian springs and flooding the spring through the river’s waters has been shown, while there was no connection to the distance of the spring from the river bed. This lack of connections from the distance was due to the yearly flooding of all riparian springs located in the valley. The lack of such a way dependence in the present research results from the hydromorphology of the Krąpiel river valley, where the springs located far away from the river bed are often flooded by rainwater flowing from the basin, and spring permanency is not related to its location in the valley. Therefore, as parameters influencing the fauna of springs, the permanence of the springs and the time of their flooding should be taken.

Our results showed that the water mite fauna of flooded springs was quantitatively and qualitatively poorer than that of non-flooded springs. In addition, flooded springs have a much lower percentage of crenobionts than non-flooded springs. This is in agreement with Von Fumetti, Dmitrović & Pešić (2017), who showed that flooding significantly influences the composition of the species assemblages of riparian springs in the valley of the River Cvrcka in Bosnia and Herzegovina, with a higher percentage of crenobiontic species in non-flooded springs (25%) than in flooded ones (13%). As a result, the presence in the flooded spring of this type of fauna indicates an advantage of the impact of spring waters over flood waters. This situation occurs in the springs from the Z6 locality, where, despite the periodic flooding of springs by the river’s waters, both crenophilous and crenobiontic fauna existed (Table 2). In opposition to this, there are the springs from the locality of Z1, where there was only a small water bodies element, resulting from the dominant influence of flood waters. Crenobiontic fauna have a much less significant dispersal ability than rheobiontic fauna and therefore the damaged fauna is reborn much more slowly even the same environmental conditions (Szlauer-Łukaszewska & Zawal, 2014; Stępień et al., 2015; Zawal et al., 2015; Zawal et al., 2016a; Zawal et al., 2016b; Zawal et al., 2016c; Zawal et al., 2017; Dąbkowski et al., 2016; Płaska et al., 2016; Buczyński et al., 2016; Pakulnicka et al., 2016a; Pakulnicka et al., 2016b; Buczyńska et al., 2017; Gerecke, Martin & Gledhill, 2017). We did not find any rhitrobiontic species in flooded springs: these were dominated by lenitobionts and to a lesser extent by crenobionts. Von Fumetti, Dmitrović & Pešić (2017) found that assemblages including water mites inhabiting riparian springs adjacent to higher-order streams were dominated by rhitrobionts. The dominance of lenitobionts in flooded springs is probably influenced by the spatial arrangement of water bodies in the landscape: there are numerous stagnant water bodies with a rich fauna in the Krąpiel valley (Stryjecki et al., 2016). All the lenitobionts found in the springs were also present in water bodies elsewhere in the valley, but in great abundance. A previous study on the water mite assemblages of water bodies in the Krąpiel valley demonstrated that the stagnant water bodies in this valley strongly influence the formation of the river fauna, in that species are thought to migrate between them (Stryjecki et al., 2016). The riparian springs lie on the potential migration route of aquatic insects from the stagnant water bodies to the river, and lenitobiontic water mites migrating as parasitizing larvae to the river can colonize springs along the way. This is also the case with springs filled with water from the nearby land (catchment area flooding). The water mite assemblage inhabiting these springs were dominated by species characteristic of temporary water bodies. In comparison with springs flooded by the river, their fauna was richer both in diversity and abundance.

Intermitent springs (Bryan, 1919) have a diverse faunistic structure (Wohltmann, 2004). The impact of intermittence on the riparian springs adjacent to a lowland river have not yet been studied. Our study showed that the water mite assemblages in these springs are dominated by lenitobionts, and that intermittency prevents crenobiontic and crenophilic water mites from occurring in intermittent springs. This concurs with an earlier study, which showed that intermittent springs were not inhabited by spring specialists (Gooch & Glazier, 1991).

The presence of fully-sclerotized specimens following the resumption of water flow in the intermittent springs confirmed the observations of some authors that water mites inhabiting unstable environments are extremely resistant to desiccation (Wohltmann, 2004). According to Viets (1923), species of the genus *Parathyas* are very resistant to the drying out of water bodies and a minimal amount of water in the moss or sludge at the bottom is sufficient to ensure their survival. Wiggins, Mackay & Smith (1980) found that deutonymphs and adults of many species occupying temporary water bodies pass the dry period buried in the sediment. Wohltmann (2004) collected postlarval instars of the water mites *Hydryphantes ruber* and *Thyas barbigera* during terrestrial phases in damp soil. The active instars of hydryphantoid water mites (*Hydryphantes*, *Thyas*, *Euthyas*) were able to crawl quite fast under terrestrial conditions, but they did not usually leave their retreat until the onset of flooding (Wohltmann, 2004).

Water mite assemblages of permanent, non-flooded springs were dominated by crenophilic and crenobiontic species. Colonization of these springs depends on the dispersal abilities of their host, not on colonization from the river (Von Fumetti, Dmitrović & Pešić, 2017). We found a higher number of rhithrobionts in non-flooded springs and all of them did not occur in the River Krąpiel. We can assume, therefore, that the colonization of non-flooded springs by water mites does not take place across the river but through the transfer by flying insects, and it is not limited by distance. This confirms previous observations (Von Fumetti, Dmitrović & Pešić, 2017).

In the Krąpiel river valley, water mites typical of springs were found in springs Z2/1, Z2/2, Z2/4, Z3/2, Z3/3, Z5/1, Z5/2 and Z5/4, which were either permanent and never not flooded, or despite periodic flooding, maintained their source character thanks to a significant supply of groundwater (springs: Z2/3, Z3/5, Z4/1, Z4/2, Z6/2, Z6/3 and Z6/4). On the other hand, sources with a poor supply of groundwater, flooded with surface waters or with a high degree of astatism (springs: Z1/1, Z1/2, Z1/3, Z3/4, Z5/5 and Z6/1) did not have a source element at all.

Our study did not reveal any impact of hydrochemical parameters on the composition of the water mite assemblages of the riparian springs studied along the Krąpiel. The lenitobiontic species, which are the dominant component of the fauna of these springs, are broadly tolerant of the majority of the physicochemical parameters of water (Cantonati, Gerecke & Bertuzzi, 2006; Smit & Van der Hammen, 2000; Davids et al., 2006; Di Sabatino et al., 2010; Gerecke et al., 2016). Von Fumetti, Dmitrović & Pešić (2017) suggested that generalist species are generally stronger competitors in flooded springs.

Several studies have suggested that factors acting at the landscape scale should be taken into consideration when analysing of spring fauna (Buczyński et al., 2003; Pakulnicka et al., 2016a; Pakulnicka et al., 2016b). The analysis of the dependence of water mite fauna on landscape parameters in this study indicates that the major factor influencing the distribution of water mites in the riparian springs was the presence in the basin of large natural or semi-natural areas (‘a mead’, ‘a forest’, ‘a shrub’, ‘a wast’, ‘a marsh’, ‘a st wat’). The large number of such areas by contrast to areas under anthropic transformations (agricultural areas, built-up areas) is characterized by the significant retention of water, which limits the flooding of the river valley and does not increase the productivity of river waters, as a result of surface runoff. The positive correlation between the area of these patches and crenobiontic and crenophilous species shows that these species are sensitive to flooding by surface waters and increased productivity of water. The negative correlation between the presence of these patches in the catchment and lenitobiontic species indicates the resistance of these species or even their preference for flood plains with increased productivity of waters. In addition, the positive correlation with the density of mixed forest patches ‘PD (13)’ and their average shape index ‘MSI (13), which is associated with increased water retention, indicates the sensitivity of spring water mite assemblages to flooding by surface waters. Surprisingly, the anthropogenic transformation of the river valley (RHM and HMS) has had a positive influence on the crenobiontic fauna. This is probably because in a modified river valley with a reinforced riverbed, springs are less often flooded by the river, which prevents the modification of the spring fauna and permits the existence of crenobiontic species. A similar effect is caused by the presence of willow shrubs near the river, which, due to the impoundment, cause longer periods of flooding in the valley, and hence a positive correlation with the distance of these patches from the shrubs ‘L (15)’. Negative correlations with the built-up areas ‘L (1)’ and ‘L (2)’ were specific cases related to the proximity of these areas to the river bed in the ‘Z6’ locality, which was rich in springs and the fauna of springs.

Water mites are an extremely useful but undervalued group of animals in terms of bioindication (Goldschmidt, 2016), but despite this they were not included in the Water Framework Directive (European Commission, 2016). When comparing the bioindicational properties of water mites and other animal groups and plants in relation to springs, we notice a very high indicator value for the water mites. This is due to the high stenotopism of the water mite species associated with the springs and the dispersal possibilities dependent on aquatic insects (Zawal, 2003; Martin & Stur, 2006; Baker, Mill & Zawal, 2008; Zawal & Szlauer-Łukaszewska, 2012).

## Conclusions

As the riparian springs in the Krąpiel river valley are mostly small and unstable ecosystems, their fauna was under great influence from external factors. The most important factor was the flooding of the springs, both by the water from the river and from the catchment. It was a degrading factor, causing an increase in crenoxenes and a reduction in crenotypic species. The periodical drying of the springs caused the settlement of populations of vernal astatic species typical of temporary water bodies, while crenotypic species were not numerous in non-permanent springs.

It seems that landscape factors do not act on springs in a distinct manner. However, river regulation prevents the frequent flooding of the valley and stabilizes the conditions in riparian springs, which allows crenobiontic species to persist. None of the physicochemical parameters of the water were statistically significant for water mite species distribution, while only one abiotic factor (sediment sorting) was statistically significant.

##  Supplemental Information

10.7717/peerj.4797/supp-1 Supplemental Information 1 Appendix S1 Database—source data.

10.7717/peerj.4797/supp-2 Supplemental Information 2 Appendix S2 Proportions of synecological assemblages in flooded and non-flooded springs.

10.7717/peerj.4797/supp-3 Supplemental Information 3 Appendix S3 Proportions of synecological assemblages in permanent and temporary springs.

## Additional Information and Declarations

### Data Availability

The following information was supplied regarding data availability: The raw data are provided in a Supplemental File.
